# Supplementary material for: Studying the context of psychoses to improve outcomes in Ethiopia (SCOPE): Protocol paper
Source: PLoS One. 2024 May 9;19(5):e0293232. doi: 10.1371/journal.pone.0293232 (PMC11081395; doi:10.1371/journal.pone.0293232)
Supplement: S1 File — (DOCX) [file pone.0293232.s001.docx]

**Supplementary File 1. Detailed description of settings**

*Rural sites:* The rural districts encompass populations with different ethnicities (predominantly Gurage and Oromo) and religions (majority Muslim populations in Misrak Meskan/Merab Meskan districts, majority Ethiopian Orthodox Christian populations in Sodo and South Sodo; mixed Protestant Christian and Ethiopian Orthodox Christian populations in Sodo Daci/Kersa Malima/Sabat Hawas). We therefore anticipate heterogeneity of help-seeking practices. There is also variation in exposure to relevant potential risk factors for psychosis: in Misrak Meskan/Merab Meskan districts, the prevalence of khat-chewing is at least 50% but alcohol use is low; in Sodo/South Sodo districts, there is a high burden of alcohol use disorders but khat use is very low.

In the rural sites, most people are subsistence farmers and/or small-scale traders. The terrain is challenging, with limited penetration of all-weather roads and a combination of hilly highland areas and lowland plains. Inhabitants of the districts are subject to chronic food insecurity, with periodic exacerbations at times of drought or unrest. Health services are largely focused on primary care: health centres are staffed by health officers, midwives and nurses serving 25,000-40,000 people, each linked to 8-10 female community-based health extension workers who are based in health posts. Hospital-based, psychiatric nurse-led out-patient services are only available in the three main towns: Butajira, Bui and Leman. An in-patient ward opened in Butajira hospital in 2021.

*Urban site:* In Addis Ababa public healthcare provision is geared towards primary care, with community-based health extension workers and health centres, but hospital-based and specialist mental healthcare services are available. The private healthcare sector is important in Addis Ababa, estimated to be used by 50% of the city population. Nonetheless, many city-dwellers live in poverty and do not readily access healthcare. There are two major Orthodox Christian holy water sites where people with psychosis are brought from Addis Ababa and where the Department of Psychiatry (Addis Ababa University) has outreach programmes [141, 142].
