# Supplementary material for: Studying the context of psychoses to improve outcomes in Ethiopia (SCOPE): Protocol paper
Source: PLoS One. 2024 May 9;19(5):e0293232. doi: 10.1371/journal.pone.0293232 (PMC11081395; doi:10.1371/journal.pone.0293232)
Supplement: S2 File — (DOCX) [file pone.0293232.s002.docx]

**Supplementary File 2: Details of formative research**

Resource mapping

Research question: what are the care options, pathways and community resources to support recovery of people with psychosis?

Previous work from Ethiopia identified community resources with potential to be mobilised for people with mental health conditions in rural districts [76]. We are using similar methodology to map out community resources in Addis Ababa, including compiling input from SCOPE advisory boards, publicly available information on community organisations, and mapping community resources in the SCOPE sub-cities using a phone-based Geographic Information System. We are also using an abbreviated version of the PRIME situation analysis tool to map out community and health system characteristics of relevance to mental health care across all study districts [77], in addition to a desk review of Ethiopian literature, and consultation with key informants and the community advisory board members to map out care pathways in rural and urban settings to inform development of identification and engagement strategies.

Ethnographic study

Research question: what are the culturally important aspects of family communication in households of people with psychosis in Ethiopia?

Study design: Ethnographic study comprising participant observation and in-depth interviews.

Sample: Ethnographic observations will be conducted in households of people with psychosis, purposively selected based on urban/rural location, trajectory of illness, and educational level of household head, recruited from the Butajira psychiatric clinic or Sodo district mental health care services (rural site), or Lideta sub-city health centres or mental health services (Addis Ababa). Key informant interviews will also be conducted with people with psychosis, caregivers, mental health care providers and community leaders (see Table 3). The final sample size will depend on when saturation is achieved, allowing comparison across settings and perspectives.

Study procedures: In-depth interviews will use a topic guide that explores patterns of family interaction, and perspectives on the impacts of mental ill-health and the status of the individual with psychosis in the family. Interviews are conducted in Amharic, transcribed and translated into English. For participant observations, a researcher will spend two hours at a time with each family, scheduled for different times of the day, to observe family members’ activities on arrival and their interactions with the person with psychosis. Each household with a person with psychosis will be observed for an estimated 30 to 40 hours over a period of six months.

Data analysis: We will use an interpretive phenomenological approach to analysing the ethnographic data, while thematic analysis will be used for the in-depth interviews [79], using NVIVO-12 software [80]. We will triangulate findings from observations and interviews.

Table 3: Formative qualitative study sample

|  | **Ethnography: households for participant observation** | **Key informant interviews** | | | |
| --- | --- | --- | --- | --- | --- |
|  |  | **People with psychosis** | **Caregivers** | **Mental health practitioners** | **Community leaders** |
| Estimated sample | 12-20 households | 20-30 interviews | 10-15 interviews | 10-15 interviews | |
| Inclusion criteria | - Person with a diagnosis of a primary psychosis (schizophrenia, schizoaffective disorder, delusional disorder and others) with no relapse in the past 6 weeks - Lives in Addis Ababa or the rural districts participating in SCOPE and has resided in that household for at least 6 months. | | Has a significant caregiving role for the individual with psychosis interviewed for SCOPE (e.g. in terms of frequency of contact and influence over care environment) | Experienced with out-patient and community-based care of people with psychosis in rural and urban settings in Ethiopia. | Is an opinion leader and has a role in shaping expectations of family communications in the community |
|  | - Willing to allow research assistant into home environment - Other household members willing to participate |  |  |  |  |
|  | - Aged 18 years or above | | | | |
|  | - Provides informed consent to participate | | | | |
| Exclusion criteria | - Unable to converse in Amharic | | | | |

Instrument adaptation and development study

*Research question:* what is the semantic, content, construct and convergent validity of newly adapted/developed measures of (a) family communication and (b) personal recovery?

*Study design:* Expert consensus meetings, cognitive interviewing, pilot study, validation study.

*Expert consensus meetings:* We are conducting online consensus meetings with international experts and in-person meetings with experts in Ethiopia, at three stages of the process, including people with lived experience of psychosis, caregivers, mental health researchers, mental health professionals, a psychometrician and statistician. Consensus meetings will be used to (1) review qualitative study findings to determine whether existing measures can be adapted or a new measure is needed[49, 144], (2) review an initial list of items to comment on comprehensiveness and content validity, (3) review the findings from the cognitive study and pilot study to agree on item reduction, (4) review validation study findings, to finalise the measures.

*Cognitive interviewing:* After measure adaptation/development, the extended list of items will be tested using cognitive interviewing with people with psychosis and caregivers to explore comprehensibility and acceptability, and make appropriate edits.

*Pilot study:* The first version of each measure will be piloted in 200 people with psychosis and 200 relatives/caregivers recruited from predominantly rural districts in south-central Ethiopia. We will follow up a sub-set of n=50 people with psychosis and n=50 caregivers two weeks after the initial study to examine test-retest reliability at the item level. The psychometric properties of the first versions of the measures will be investigated, including item-item and item-total correlations, internal consistency (Cronbach’s alpha), exploratory factor analysis to investigate the dimensional structure of the scales, and Kappa coefficient for test-retest reliability. The psychometric properties of the items and their conceptual relevance will inform item reduction to produce the second version of the measures.

*Validation study:* The second version of the measures will then be tested in a sample of 400 people with psychosis and 400 relatives/caregivers attending psychiatric out-patient clinics in Addis Ababa. This sample size will be sufficient for us to carry out confirmatory factor analysis and use item response theory to examine the performance of items, including differential item functioning by gender. In addition, we will investigate convergent validity with symptom severity (measured using the Brief Psychiatric Rating Scale, extended version[145]) and functional impairment (measured using the validated World Health Organization Disability Assessment Schedule, version 2.0[96]). The final contextually valid measures of family communication and personal recovery will be used in the baseline of the epidemiological study.
