# Supplementary material for: Studying the context of psychoses to improve outcomes in Ethiopia (SCOPE): Protocol paper
Source: PLoS One. 2024 May 9;19(5):e0293232. doi: 10.1371/journal.pone.0293232 (PMC11081395; doi:10.1371/journal.pone.0293232)
Supplement: Attachment — Submitted filename: Response to PLOS One.docx [file pone.0293232.s005.docx]

Dear Editor,

I have reviewed the response of the authors and their revision of the manuscript and found them to be satisfactory.

I recommend accepting the manuscript for publication.

Regards.

Olatunde O Ayinde.
