# Supplementary material for: Studying the context of psychoses to improve outcomes in Ethiopia (SCOPE): Protocol paper
Source: PLoS One. 2024 May 9;19(5):e0293232. doi: 10.1371/journal.pone.0293232 (PMC11081395; doi:10.1371/journal.pone.0293232)
Supplement: Attachment — Submitted filename: Reviewer comment on SCOPE manuscript..docx [file pone.0293232.s003.docx]

Reviewer’s comments for Studying the context of psychoses to improve outcomes in Ethiopia (SCOPE): protocol paper for PLOS ONE

This programme of work is an important addition to several others being carried out in the Global South aimed at understanding the nature of severe mental disorders and meeting the needs of those affected by these conditions within the cultural and social context of where they reside. It builds on the rich tradition of psychosis research in Ethiopia and extends the ongoing efforts in that country to expand services for persons living with psychosis. It has the potential of providing a model on which other countries across the developing world can pattern their aspirations of increasing access to nuanced mental health services to numerous individuals affected by psychosis and their families in the different settings. Below are some areas in the manuscript requiring clarification:

1. Eligibility criteria for SCOPE epidemiological study: the authors wish to reduce the lower limit of eligibility age to 15 years. It is important that the investigators provide some reason/ explanation for this decision to extend the lower age limit of their sample to 15 years and comment on the ethical implications of this decision, and how they wish to address it, in the manuscript.
2. For the purpose of SCOPE, how do the investigators define “homelessness”? The concept of homelessness can mean different things depending on whether it is being discussed in within Western or African cultural contexts, and may also have urban-rural differences in meaning. It is important to include this definition in the manuscript, even if briefly.
3. Page 18, line 420. I suspect you mean “test-retest”, not “test-restest”.
4. Page 21, line 460-462: “Data collection will be paper based for some aspects of the semi-structured measures, but mostly electronic (using smartphones or tablets) for structured interviews and stored securely on the **university** server.” Which university are the authors referring to?
5. Ethical considerations (page 23). While the authors have provided extensive information on the process of obtaining consent on the field, it does happen from time to time that research staff on the field are unsure whether some potential respondents have capacity to consent or not. Could the authors comment on how this will be resolved if and when it occurs? Will senior investigators be contacted to resolve uncertainties in the capacity of potential respondents to give consent? Will another mental health practitioner unconnected with the study be employed to determine capacity to consent if the authors wish to be mindful of conflict of interest?
6. Formative qualitative study: Regarding the ethnography component of the study, entire households will be observed by the investigators. How do the authors wish to approach consent? Will this be from the head of the household or each member of the household? What happens some members of the household do not give consent for ethnographic study, when the head of the household has?
